# *Saccharomyces cerevisiae* Linker Histone—Hho1p Maintains Chromatin Loop Organization during Ageing

**DOI:** 10.1155/2013/437146

**Published:** 2013-08-19

**Authors:** Katya Uzunova, Milena Georgieva, George Miloshev

**Affiliations:** Laboratory of Yeast Molecular Genetics, “Acad. Roumen Tsanev” Institute of Molecular Biology, Bulgarian Academy of Sciences, “Acad. G. Bonchev” Street, Building 21, 1113 Sofia, Bulgaria

## Abstract

Intricate, dynamic, and absolutely unavoidable ageing affects cells and organisms through their entire lifetime. Driven by diverse mechanisms all leading to compromised cellular functions and finally to death, this process is a challenge for researchers. The molecular mechanisms, the general rules that it follows, and the complex interplay at a molecular and cellular level are yet little understood. Here, we present our results showing a connection between the linker histones, the higher-order chromatin structures, and the process of chronological lifespan of yeast cells. By deleting the gene for the linker histone in *Saccharomyces cerevisiae* we have created a model for studying the role of chromatin structures mainly at its most elusive and so far barely understood higher-order levels of compaction in the processes of yeast chronological lifespan. The mutant cells demonstrated controversial features showing slower growth than the wild type combined with better survival during the whole process. The analysis of the global chromatin organization during different time points demonstrated certain loss of the upper levels of chromatin compaction in the cells without linker histone. The results underlay the importance of this histone for the maintenance of the chromatin loop structures during ageing.

## 1. Introduction

Ageing consists of natural alterations in the cells which are implemented by molecular programs written in the genome and in the epigenome at the same time. The organization of DNA in chromatin enables the epigenetic information transfer to nuclear processes. The first level of chromatin organization, the nucleosome arrays, consisting of DNA wrapped around nucleosomes is relatively well known [1]. Several lines of evidence have shown that the basic epigenetic role of chromatin in ageing is accomplished at this particular level of chromatin organization [2–5]. The upper levels above the nucleosome arrays, known as 30 nm chromatin fibers, chromatin loops, chromosome territories, and so forth, are yet far from being comprehensibly described [4, 6]. Their part in the epigenetic inheritance is barely known though it was proved that the main participants in the building and preservation of these structures are members of the family of linker histones—H1 [7–9].

Although data about the epigenetic transformations of chromatin during cellular ageing has been accumulated extensively in the recent years, information about age-related higher-order chromatin alterations is practically nonexistent. A good exception is the study showing that loss of two proteins, PBBP4 and PBBP7, subunits of the NURD chromatin remodeling complex, compromises the establishment and maintenance of the higher-order chromatin structure, thus possibly making chromatin more susceptible to DNA damage [10].

A brilliant model for ageing research is the yeast *Saccharomyces cerevisiae* [11]. Many stages and molecular signatures of ageing such as accumulation of oxidative damaging, involvement of mitochondria in the process, and connection with the nutrient response pathways have been revealed in this unicellular eukaryote [12–16]. At the same time the yeast cells are good models for chromatin research, mainly for studying the roles of linker histones. In contrast to the multiple linker histone subtypes in the higher eukaryotic cells there is only one-copy gene (*HHO1*) for linker histone, Hho1p in *S. cerevisiae* [17]. Interestingly, though the gene is non-essential, its disruption leads to severe alterations in the higher-order chromatin structures during somatic growth, thus stating the need for further analyses of the exact roles and functions of linker histones during different molecular processes [18]. 

In the current study we searched for the roles which *S. cerevisiae* linker histone—Hho1p, could play in the propagation of the ageing process. The gene for this histone in yeast cells has been knocked-out and thus, linker histone-free strains were developed as suitable models for chromatin studies during cellular ageing. Our results show that the yeast cell cultures devoid of linker histones had slower growth in minimal media accompanied by well-demonstrated delay during their logarithmic growth. Moreover, the cells showed an increased survival rate during the whole period of cultivation. Studies on the overall chromatin organization of these mutant cells uncovered the existence of longer chromatin loop sizes and distorted higher-order chromatin organization accompanying the ageing process. Taken together our results underlay the importance of the linker histones and the higher-order chromatin structures in cellular ageing.

## 2. Materials and Methods

### 2.1. Yeast Strains and Growth Media

*Saccharomyces cerevisiae* strains used in the current study were derived from two genetic backgrounds DY2864 and FY1679-08a. The deletions of the gene for the linker histone *HHO1* were done according to the technique of [19]. The disruption cassette *hho1Δ::KlURA3* bore *URA3* gene from *Kluyveromyces lactis* flanked by two long sequences homologous to the outer ends of the chromosomal copy of *HHO1* gene [18]. The genotypes of both the wild type and *hho1delta* strains are listed below.

#### 2.1.1. DY2864

*DY2864 (wt)*: MATa his4-912*δ*-ADE2 his4-912*δ* lys2-128*δ* can1 trp1 ura3 ACT3.

*DY2864 (hho1delta)*: MATa his4-912*δ*-ADE2 his4-912*δ* lys2-128*δ* can1 trp1 ura3 ACT3 ypl127C::K.L.URA3.

#### 2.1.2. FY1679-08a

*FY1679-08a (wt)*: MATa ura3-52/ura3-52 trp1Δ63/TRP1 leu2Δ1/LEU2 his3Δ200/HIS3 GAL2/GAL.

*FY1679-08a (hho1delta)*: MATa ura3-52/ura3-52 trp1Δ63/TRP1 leu2Δ1/LEU2 his3Δ200/HIS3 GAL2/GAL ypl127C::K.L.URA3.

Chronological ageing assays were performed in SD media (1.7% yeast nitrogen base without amino acids and 2% glucose), supplemented with 20 *μ*g/mL of the appropriate nutritional requirements according to the genotype of the strains. Cultivation was at 30°C in a water bath shaker for a period of twenty days. Additionally, monitoring of CLS of the studied yeast strains was done in rich media YPD, containing 1% Yeast extract, 2% Peptone and 2% Dextrose at 30°C.

### 2.2. Chronological Lifespan Assays

Assessment of cell growth in SD media was done by taking aliquots from the yeast cultures at eight different time points, indicated as 0th (assigned as initial), 1st, 3rd, 6th, 9th, 12th, 14th, and 19th days for subsequent spectrophotometric measurement of OD_600_ (Optical Density at 600 nm wavelength) together with counting the number of cells in a Nuebauer counting chamber. Quantitative measurements of colony forming units (CFUs) were also performed. 20 *μ*L aliquots of the cultures were removed at 1st, 3rd, 5th, 9th, 12th, 14th, and 19th days diluted in sterile water, spread onto YPD plates (100 colonies per plate), and allowed to grow into colonies for 2 days at 30°C. The colonies were then counted and the number of CFUs per plate calculated. Percentage viability was calculated as in [20].

Three independent repetitions of all experiments were performed and the results were statistically analyzed by Students *t*-test at Microsoft Excel 2010 software.

### 2.3. Chromatin Structure Studies by Chromatin Comet Assay (ChCA)

Chromatin loop organization was studied by the method of Chromatin Comet Assay (ChCA) [18, 21]. 300 *μ*L from the chronologically ageing yeast cultures was gathered by centrifugation at 10 000 g for 1 min (Eppendorf microfuge). After washing in S-buffer (1 M Sorbitol, 25 mM NaH_2_PO_4_; pH 6.5) the cells were diluted to 10^5^ cells/mL with the same buffer supplemented with appropriate concentration of the spheroplasting enzyme Zymolyase (Saikagaku Corp.). The cells were immediately mixed with 1.4% of low-gelling agarose (Sigma type II) and spread like microgels onto microscopic slides by the means of coverslips. Five min incubation at 10°C followed for solidification of the agarose-cell-gel suspensions and after removing the coverslips, the slides containing the microgels were processed for spheroplasting at 37°C for 15 min. All subsequent procedures were performed in neutral conditions as follows: after solidifying microgels and subsequent *in situ* nuclease digestion of spheroplasts slides were submerged in neutral lysis solution (146 mM NaCl; 30 mM EDTA; 10 mM Tris-HCl and 0.1% N-lauroylsarcozine, pH 7.5) for 20 min in a cold room at 10°C. Afterwards slides were washed for 15 min in 0.5 x TBE buffer (89 mM Tris; 89 mM Boric-acid; 5 mM EDTA, pH 8) and subjected to electrophoresis for 10 min at 0.45 V/cm in the same TBE buffer. After successive dehydration in 75% and 100% ethanol for 5 min each, the slides were left to air-dry.

Comets were observed under a Leitz epi-fluorescence microscope (Orthoplan, VARIO ORTHOMAT 2) using 450–490 nm band-pass filter following the staining of DNA with SYBR green I (Molecular Probes Inc, Eugene, OR, USA). Pictures were taken with a digital camera, Olympus *μ*800, at a resolution of 3 mpx. Images were bright-contrast processed using Adobe Photoshop CS 5.1. software.

The obtained results were quantified by measuring the parameter Comet length on Adobe Photoshop CS 5.1 and then calculated in kilobases as was published elsewhere [18].

Two independent repetitions of the ChCA experiments were done and results were analyzed statistically by GraphPad Prism 3.0 software.

## 3. Results and Discussion

### 3.1. The Lack of *Saccharomyces cerevisiae* Linker Histone Leads to Slower Growth Rate during CLS and at the Same Time to Increased Survival

**Figure 1:**
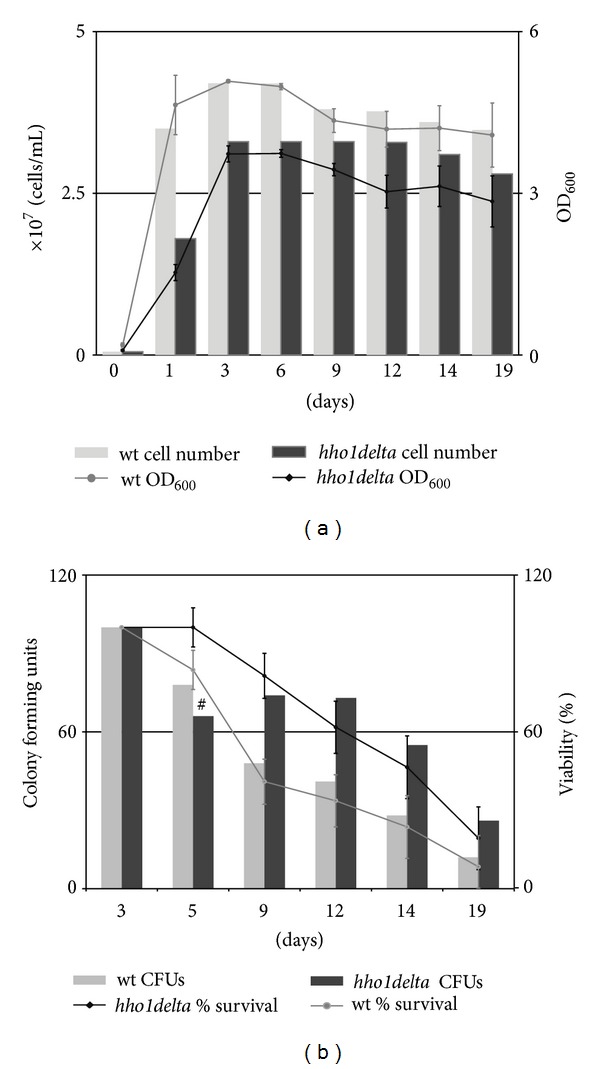
The lack of *S. cerevisiae* linker histone leads to slower growth rate during CLS and increased survival. (a) Yeast wild type and *hho1delta* cells were cultured in SD media for a period of 20 days. At certain time points aliquots were taken for spectrophotometric OD_600_ measurements and for counting the number of cells in a Nuebauer counting chamber. Results are presented as combined graphs comparing at one time the growth rate (lines) and the absolute number of cells at each time point (bars). Every time point stands for three independent repetitions of the experiments with STDVs (±). The difference between the wild type and *hho1delta* culture growth were statistically significant (*P* < 0.01). (b) The survival rate of *hho1delta* mutant cells and their progenitor wild type strains was assessed by plating 10^2^ cells on YPD plates at every two days during the CLS 30°C for two days allowing cells to divide. CFUs were then counted and percentage survival was calculated by assuming the number of CFUs for each strain at 3rd day for 100% (lines). Additionally, the number of colonies was included in the graph (bars) thus allowing easy and comprehensible comparison between the CLS of wild type and *hho1delta* cells. ^#^ *P* > 0.01 was statistically insignificant only for CFUs at 5th day. At all other time points the differences between the wild type and *hho1delta* cultures were significant *P* < 0.01 and for the simplicity of the graph presentation are not marked on the figure.

Chronological lifespan of *S. cerevisiae* wild type and *hho1delta* strains were studied in SD media, recommended as an appropriate media for monitoring CLS of yeast cultures [20, 22] for a period of 20 days at 30°C. CLS assays were performed as cultures' optical densities were followed by spectrophotometric measurements at 600 nm wavelength (OD_600_) and by counting the number of cells at certain time points during the whole period of cultivation. The obtained results were plotted as combined chart type graphs representing at one time the cultures' OD_600_ (lines) and the absolute number of cells counted in a Nuebauer counting chamber (bars) (Figure 1(a)). The time points selected for monitoring of the growth rate of the cultures were day 0 (assigned as initial or a dilution day), 1st day as a day representing the logarithmic phase of the cultural growth, 3rd day as a day marking the post diauxic phase, 6th day as the beginning of the stationary phase, 9th, 12th, 14th and 19th day as these four days strongly represent the stationary phase of our cultures. These particular time points were picked up according to other authors' data depicting the different stages of yeast CLS summarized in [20]. As seen in Figure 1(a) the mean OD_600_ for the wild type was 4 and for *hho1delta* cultures only 2.6; thus, *hho1delta* cells demonstrated 35% slower mean growth than the wild type. Moreover, *hho1delta* cells demonstrated an inability to reach the highest optical density of the wild type cultures but rather reached only 73% of the highest value of wild type OD_600_ at 3rd day (5.08). The number of cells (cells/mL) counted separately for each time point ascertained these results. Furthermore, they allowed quantification of the growth rate of the yeast cell cultures. Obviously, both the wild type and the mutant reached the highest OD_600_ at 3rd day; however, the mutant had fewer cells. These differences were statistically significant and highlighted the importance of the linker histone during yeast CLS in SD media. Moreover, the mutant cells had an expressed lag phase during its logarithmic growth seen on 1st day with the estimated mean OD_600_ = 1.5 for the mutant and mean OD_600_ = 4.6 for the wild type. Note that these differences appear regardless that both, the wild type and the mutant, had the same starting cell number (10^6^ cell/mL) at the initial day. The dividing potential of *hho1delta* cells seemed somehow impeded in comparison to the normally growing wild type cultures. These data prompted a role of the yeast linker histone in the chronological lifespan and are in unison with the results of Downs et al. [23] demonstrating that cells lacking Hho1p have reduced replicative lifespan. Besides, for quite a long time in contrast to core histones the roles of linker histones in chromatin biology, ageing, and metabolism studies were merely neglected. Our results showing that Hho1p is required for the normal cellular growth in synthetic defined media together with recent data demonstrating Hho1p roles in chromatin compaction during stationary phase and moreover during overall somatic growth [18, 24] start adding new hues on the roles of this linker histone and probably other linker histones in the processes of ageing and longevity.

The CLS studies on the yeast cells lacking the gene for the linker histone continued with probing the cellular viability of these cells during the chronological lifespan. At every second day aliquots were taken from the growing cultures and 10^2^ cells were plated on YPD plates, which were further incubated at 30°C for two days. CFUs were counted and percentage survival was assessed for each strain independently. Results are plotted as combined chart type graphs simultaneously representing the percentage of survival (lines) and the absolute number of colonies (bars) (Figure 1(b)). Surprisingly, *hho1delta* mutants exhibited better survival rates than the wild type. Notably, this better survival of the mutant was accompanied by higher heterogeneity in the obtained values suggesting higher diversity in the mutant cellular populations. We assume that this diversity probably can lead to different modes of cellular response toward environmental stress conditions during the chronological lifespan. Such stress conditions can be induced by ROS and acetic acid appearing during the processes of culturing [25]. This line of thoughts necessitates further studies in the field. Up until now our results show complicated and yet not so well-understood functions of the yeast linker histone in the chronological ageing. On one hand, the mutant grows more slowly, but at the same time some of its cells survive better than the wild type. These results prompt a complex way of survival of the cells without the linker histone, probably due to one of the main functions of Hho1p, namely, its participation in the maintenance of the genomic stability [23].

Well known in the field of the yeast ageing research is the fact that the switch between fermentation and respiration is accompanied by total rearrangement of the overall cellular and genomic programme of the yeast cells [26, 27]. Therefore, noting the differences between the wild type and the mutant during cellular growth in minimal media and the diverse modes of cellular survival in the mutant background we speculate that Hho1p is an epigenetic player in gene expression, participating in the switching on and off of specific genes during stationary phase. This hypothesis is quite logic but is not yet proved and needs more experiments in the future. High-throughput microarray analyses of *hho1delta* mutants revealed that the expression pattern of only 27 of all 6000 yeast genes has been slightly changed because of the mutation. However, important is the fact that these studies have been done in rich YPD media and thus do not correlate with cellular longevity studies of yeast in minimal media [28]. Moreover, even the authors of the above study have suggested a continuation of their work with the culturing of yeast *hho1delta* mutants under different conditions and then following changes in the overall gene expression programme. 

**Figure 2:**
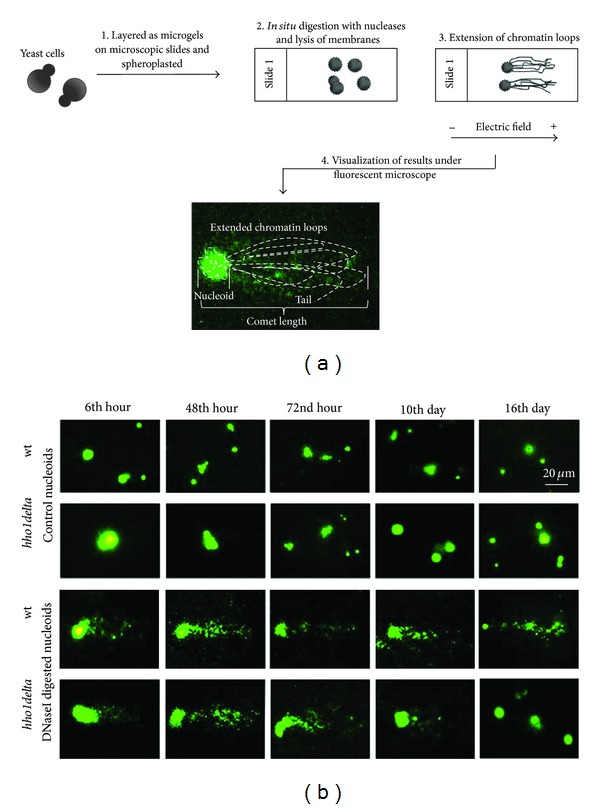
Chromatin Comet Assay (ChCA) performance and the logic behind its results. (a) A scheme of the method of (ChCA) is presented with subsequent crucial steps marked with numbers. The method is a modification of the conventional Comet Assay technique and allows easy and fast visualization of higher-order chromatin structures at a single cell level. (b) Representative images of wild type and *hho1delta* mutant comets. The white bar stands for 20 *μ*m.

In order to check whether the lack of the yeast linker histone leads to abnormalities in the ageing process in rich media, we incubated the studied yeast strains in YPD. The results are shown in Figures 1S and 2S (see Supplementary Materials available online at: http://dx.doi.org/10.1155/2013/437146) representing that there is no significant difference between the wild type and the deletion mutants. Growth rates (Figure 1S) and percentage survival (Figure 2S) demonstrated almost the same CLS of the studied cells in YPD which is in unison with the results of other authors presenting slightly changed gene expression patterns in *hho1delta* cells in the same media [28].

Regarding our data showing slower cellular growth and increased percentage survival of the knocked-out yeast cells in SD media in a time course of almost 20 days we decided to check whether Hho1p takes part in the building and maintaining of the higher-order chromatin structures during CLS. As chromatin is the platform on which all processes on the molecule of DNA take place, it is easy to make the assumption that higher-order chromatin structures participate in the processes of ageing. Therefore, we followed the way by which chromatin structure was changing during chronological lifespan.

### 3.2. Chromatin Comet Assay (ChCA) as a Powerful Tool for Monitoring Higher-Order Chromatin Organization during Ageing

As the method of Chromatin Comet Assay is not very common in ageing research, here we feel that some detailed explanations of how it is performed and what it studies are needed. The method has been developed with several modifications of the standard Comet Assay [29, 30]. The crucial point was to determine the optimal conditions for sensitive and correct visualization of the electrophoretic extension of chromatin loops from the nucleus after mild nuclease digestion (Figure 2(a)). The nucleases that we use in performing the method are MNase (micrococcal nuclease) and DNase I (deoxyribonuclease I), but any kind of DNA cutting enzyme can give reproducible results although with different meaning. Briefly, yeast cells are mixed with a spheroplasting enzyme, in our case Zymolyase, then with low-gelling agarose and are spread as microgels on microscopic slides. After 15 min of spheroplasting at 37°C the microgels are digested *in situ* with the nucleases and again are incubated for 1 min at 37°C. This step allows mild cuttings of chromatin which is dependent on the way chromatin is organized and compacted in the nucleus. A schematic drawing of the ChCA is shown in Figure 2(a) and allows easy following of the main steps of the methodology. The *in situ* nuclease digestion is followed by lysis of cell membranes, resulting in the turning of spheroplasts into nucleoids. During the next step, the electrophoresis, under the electric field chromatin loops from the nucleoids protrude toward the anode and thus form a comet-like image (Figure 2(a)). Note that because of the mild nuclease digestion the comet tail consists of chromatin loops, that is, chromatin structures with high molecular weight, around 200–300 kb in size [18, 21], which is far bigger than the DNA fragments obtained in experiments for assessing nuclease sensitivity reaching to nucleosome ladders. This is the reason for accepting ChCA as a method for higher-order chromatin structure studies, more precisely for chromatin loop structure monitoring at a single-cell level. The cellular comets contain a head which stands for the DNA with an intact structure and a tail extended toward the anode. In the tail are the chromatin loops which are relaxed due to several cuts in DNA. Different ways of Comet Assay data quantification exist including measurement of the length of the comet, the length of the comet tail, the intensity of DNA in the tail, and/or the intensity of DNA in the head. When ChCA is performed these parameters are used for quantitative data analyses, but more often the length of the tail and the length of the comet are measured on image processing software programmes and after that are calculated in kilobases in order to estimate the chromatin loops length [21].

### 3.3. The Yeast Linker Histone Maintains Chromatin Loop Structures during Chronological Lifespan

The above experiments on wild type and *hho1delta* yeast cells demonstrating the way the mutant and its progenitor wild type strain grow and survive through their CLS allowed us to choose time points for further analyses by Chromatin Comet Assay. We have chosen five time points: the 6th hour, the 48th, the 72nd hour, and 10th and 16th days. Yeast cells taken at these time points were further used for the ChCA and aimed to reveal chromatin loop structure reorganization during ageing. The nuclease used during these particular ChCA experiments was DNase I. Generally, it makes single-stranded DNA cuts with a preference to active chromatin domains [1] and thus allows relaxation of chromatin loops and probing spatial chromatin organization. Representative images of ChCA for each strain are given in Figure 2(b). Control nucleoids for both strains at the studied time points are presented together with chromatin comet images. The wild type nucleoids had a mean diameter around 20 *μ*m while the mutant nucleoids appeared 1.6 times more swollen than the wild type (33 *μ*m mean diameters for *hho1delta* controls). The performed analysis of Variance (GraphPad Prizm software) proved that this increase in the mean diameters of the mutant nucleoids is significant (*P* < 0.01), thus arguing for more relaxed chromatin organization in the yeast nuclei lacking the linker histone. Furthermore, the wild type increased their nuclear diameters with ageing to 40% larger diameter in comparison with the beginning of their CLS (from 1st day—mean diameters 19 *μ*m till 16th day—mean diameters 30 *μ*m). On the contrary the mutant cells rearranged their nucleoid size in a reverse order (Figure 2(b), *hho1delta* controls). Their diameters shrank slightly from 36 *μ*m at 1st day to 25–29 *μ*m at 16th day and got closer to the diameters of the wild type nuclei at the last days of ChCA studies 16th. This is arguing for loss of normal nuclear structure along the ageing process. Moreover, in the mutant cells at 3rd day (72nd hour) we had a sharp drop in the diameters of the nucleoids (down to 10 *μ*m) which strengthens our hypothesis that this particular time point is crucial for the mutant cells and marks total rearrangement in the genome and the expression of specific genes for adaptation of these cells to the switch between the postdiauxic phase and the stationary phase. Further studies are required to clarify these changes in order to explain the role of linker histone in the yeast chronological ageing. 

Chromatin loop structures were further analysed by digestion of the nucleoids with DNase I. With the time of cultivation up until the 3rd day both the wild type and *hho1delta* chromatins demonstrated an increase in the length and the intensity of the comets. Markedly, the dynamics of chromatin loop structures, that is, the appearance and the length of the obtained comets, for the two strains demonstrated some differences, though from the 6th till the 48th hours the wild type chromatins exhibited looser and more susceptible to the action of DNase I chromatin. This is expected having in mind that with the time of the ageing process chromatin starts losing its normal characteristics and becomes more relaxed (Figure 2(b), WT DNase I), and the logarithmically growing mutant cells (6th and 48th hour) produced longer comets than the wild type Figure 2(b). This proved that more chromatin loops were relaxed and extended form the mutant nucleoids than in the wild type, which is in good correlation with other data showing that the yeast linker histone is necessary for chromatin compaction during the overall growth of these cells and its lack leads to total rearrangement of chromatin loop structures making them less compacted and more susceptible to the action of nucleases [18, 31]. Surprisingly, though, after the 72nd hour (3rd day) till the last 16th day the mutant comets started to decrease in size till 16th day when empty and faintly visible nuclear “shades” appeared. This suggests total loss of chromatin structure. On the other hand, the decrease in the comet lengths in the wild type at these particular time points was stable and did not lead to total disappearance of the comet images, nor to observation of nuclear “shades.”

**Figure 3:**
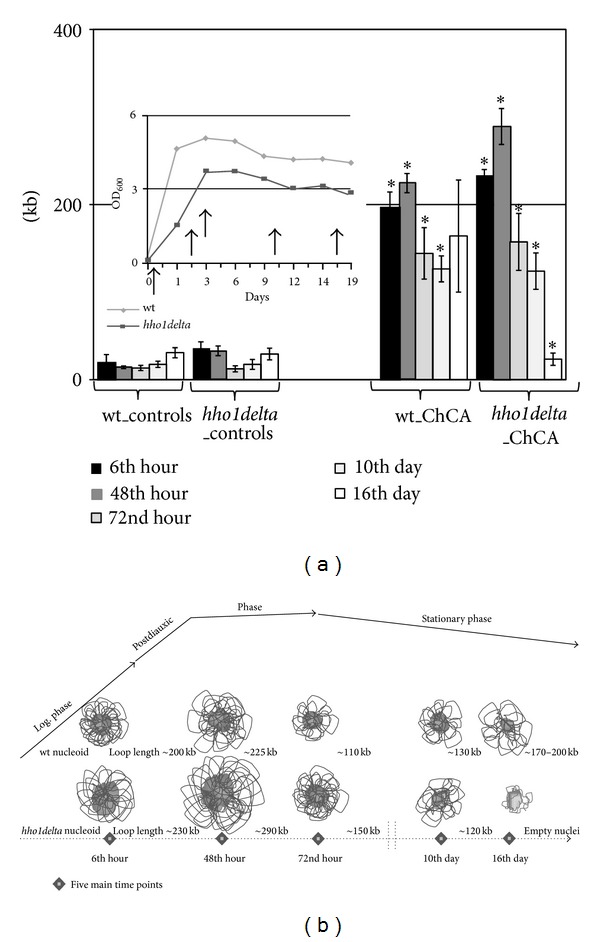
Yeast linker histone maintains and preserves the higher-order chromatin structure organization during chronological ageing. Yeast mutants lacking the linker histone were subjected to ChCA and were compared to the wild type. Chromatin structure was studied at five time points designated as main during the CLS of yeast cells in SD media. (a) ChCA results quantification-measurement of comet length and calculation of chromatin loop lengths in kilobases. The five studied time points are marked on the growth curve built in the figure. The results are presented as bars showing the mean comet length values ± STDVs. The statistical analyses proved these results as significant *P < 0.01. (b) A model describing changes in the higher-order chromatin organization during chronological ageing. On the time course of the chronological lifespan of yeast cells in minimal media are marked five main time points (diamond shape) and their reference to the CLS phases. Wild type and *hho1delta* nucleoids are drawn with the hypothetical chromatin loop organization changes during ageing. The lack of the linker histone totally abolishes normal chromatin ageing and thus influences overall cellular behaviour during the process.

ChCA data quantification was done by measuring the length of the whole comet and sizing of chromatin loops as published in [18]. Results are shown in Figure 3(a) and demonstrate 12% increase in wild type comet lengths till the 48th hour when cells were already in postdiauxic phase. Then during stationary phase the wild type chromatin became more disordered resulting in more compacted and less susceptible to DNase I chromatin organization. ChCA calculations showed that the chromatin of logarithmically growing wild type cells was organized in loops with approximate length of 200 kb which slightly increased during the postdiauxic phase extending to 225 kb, then decreased to 100–130 kb at the 72nd hour and the 10th day, probably as a result of the adaptation of cells to stationary phase. This adaptation requires activation of specific genes necessary for proper adaptation of cells from fermentation to respiration when cultured in minimal media [32]. At the 16th day we observed higher heterogeneity but the size of the chromatin loops was around 170–200 kb.

Cells without the linker histone demonstrated much more disordered chromatin organization on the first day. At the time when cells set off their logarithmic growth the comet length, that is, the size of the extended chromatin loops was around 230 kb slightly bigger than the wild type (Figures 3(a) and 3(b)). This slight 10% increase in the comet length suggests a more relaxed, less compacted chromatin in the lack of the linker histone. Previous chromatin studies of logarithmically growing *hho1delta* cells are correspondingly consistent with these observations [18]. With the entry of cells in postdiauxic phase, that is, at the 48th hour of cultivation, the difference in chromatin loop organization between *hho1delta* mutant and the wild type increased to 25%. This shows that with the ageing mutant cells reorganized their chromatin structures in a more relaxed manner allowing extension of longer chromatin loops (290 kb in length). The switch between the post-diauxic phase and the stationary phase (72nd hour and 10th day) demonstrated a drop in the comet length to 150–120 kb. This tendency kept on going leading to empty nuclei and nuclear “shades” at the end of the culture.

Chromatin is the background and the driving force for activation and deactivation of genetic information and definitely should be part of the ageing process [33, 34]. Little is known about the higher-order chromatin organization and dynamics during ageing which makes our results informative for the dynamics in these chromatin structures during this process. The presumptive model drawn on Figure 3(b) gives a comprehensible picture of general chromatin loop organization during ageing. This model allows explanation of the differences which were found between the wild type and the cells without linker histone. Some authors [35] have demonstrated roles of linker histones during senescence by showing that they are lost with the time of the ageing of cells. With our ChCA results of chronologically ageing yeast cells with and without the linker histone we were able to follow the nuclear and chromatin dynamics along the ageing process.

## 4. Conclusions

Our study has unveiled the role of the yeast linker histone Hho1p in the preservation and maintenance of the higher-order chromatin structures during ageing. Therefore, we think that Hho1p participates in the regulation and governing of the CLS of yeast and can be an active epigenetic player in cellular adaptation during this process. It is involved in the switch between logarithmic growth and the postdiauxic phase and assures the preservation of the genomic stability. These results highlight the epigenetic significance of the linker histones in holding the genomic resilience against stress and in preserving the normal nuclear morphology. Moreover, this study marks the potential of linker histones as compensatory epigenetic players in genetic disorders including syndromes of premature ageing like Hutchinson Gilford Progeria.

## Supplementary Material

Supplementary information contains: 1) a short description of the experimental procedures and 2) two figures with a title and a short description.
