# Supplementary material for: Saccharomyces cerevisiae Linker Histone—Hho1p Maintains Chromatin Loop Organization during Ageing
Source: Oxid Med Cell Longev. 2013 Aug 19;2013:437146. doi: 10.1155/2013/437146 (PMC3760111; doi:10.1155/2013/437146)
Supplement: Supplementary file 1 — Supplementary information contains: 1) a short description of the experimental procedures and 2) two figures with a title and a short description. [file 437146.f1.docx]

SI Guide

**1. Supplementary methods:**

*Cellular growth and percentage survival analysis in rich YPD media:*

Yeast wild type and hho1delta mutants were grown in rich YPD media for a period of 14 days at optimal growth conditions (30^◦^C). At certain time points aliquots were taken and cellular growth and survival percentage were checked. Cellular growth was followed by spectrophotometric measurement of Optical Density at 600 nm wave length while survival potential was assessed by plating 100 cells on solid YPD media and after two days of cultivation live cells were counted and percentage of survival calculated. Results are shown on Figure 1S for the OD_600_ measurements and on Figure 2S for the cell survival assays.

**2. Supplementary figures titles and short descriptions:**

*Figure 1S.* *Cellular growth rate of wild type and hho1delta cells in rich (YPD) media.*

Cells were growth in YPD media for a period of 14 days. At certain time points aliquots were taken for spectrophotometric analysis of the growth rate. Results are presented as mean OD_600_ for each time point and each cell culture plotted as growth curves.

Three independent repetitions of the experiment were done and results were statically elaborated, showing that the differences in the percentage survival in YPD between the wild type and *hho1delta* cells were not significant (p>0.01).

*Figure 2S.* *Percentage survival of wild type and hho1delta cells in rich (YPD) media.*

Quantitative measurements of colony forming units (CFUs) were performed as 20 μl aliquots of the cultures were taken at days 1^st^, 3^rd^, 6^th^, 9^th^, 12^th^ and 14^th^, diluted in sterile water, spread onto YPD plates (100 cells per plate) and allowed to grow into colonies for 2 days. The colonies were then counted and percentage survival per plate calculated. Results were not statistically significant # p > 0.01.
