# Supplementary material for: Saccharomyces cerevisiae Linker Histone—Hho1p Maintains Chromatin Loop Organization during Ageing
Source: Oxid Med Cell Longev. 2013 Aug 19;2013:437146. doi: 10.1155/2013/437146 (PMC3760111; doi:10.1155/2013/437146)
Supplement: Supplementary file 2 [file 437146.f2.pdf]

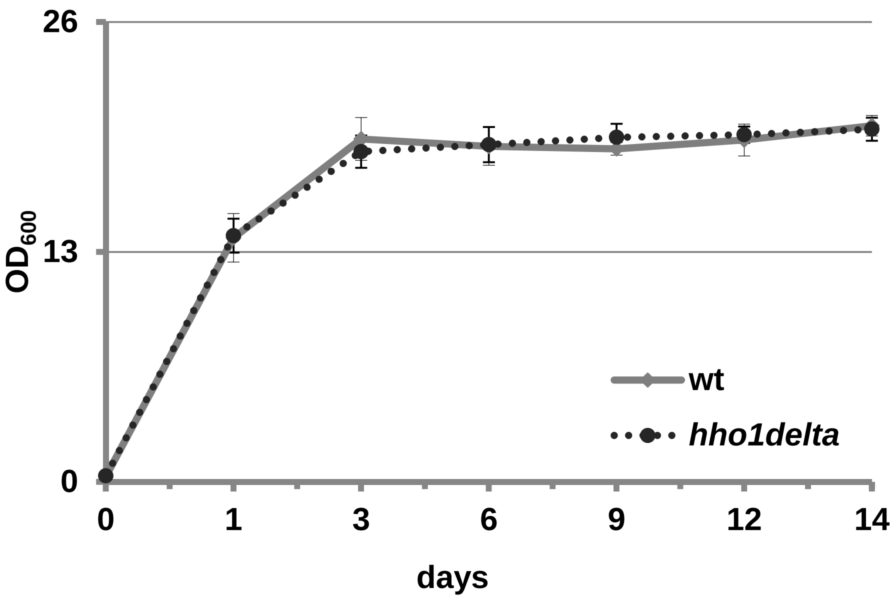

Figure 1S. Cellular growth rate of wild type and *hho1delta* cells in rich media.

Cells were growth in YPD media for a period of 14 days. At certain time points aliquots were taken for spectrophotometric analysis of the growth rate. Results are presented as mean OD_{600} for each time point and each cell culture plotted as growth curves.

Three independent repetitions of the experiment were done and results were statically elaborated, showing that the differences in the percentage survival in YPD between wild type and *hho1delta* cells were not significant ($p>0.01$).

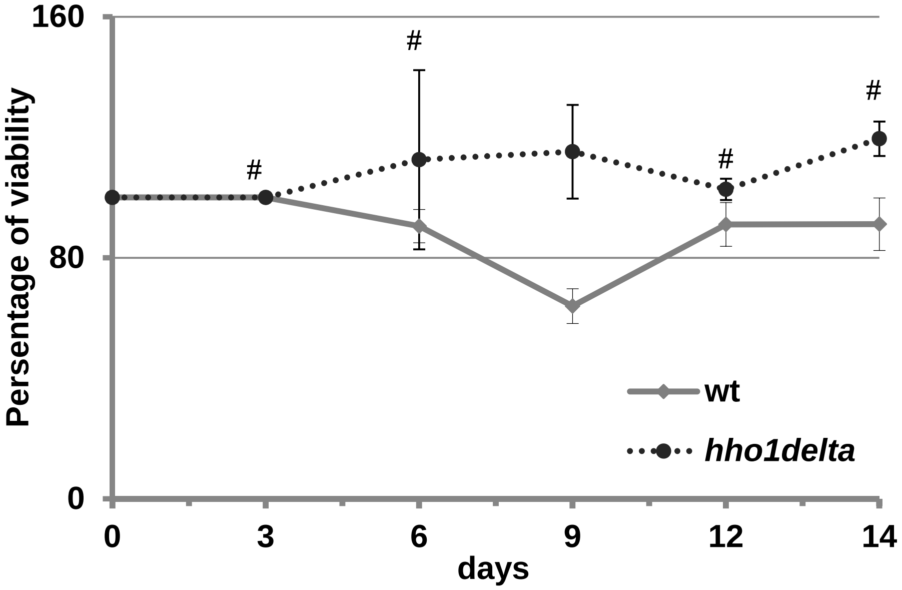

Figure 2S. Percentage survival of wild type and *hho1delta* cells in rich media.

Quantitative measurements of colony forming units (CFUs) were performed as 20 µl aliquots of the cultures were removed at days 1st, 3rd, 6th, 9th, 12th and 14th, diluted in sterile water, spread onto YPD plates (100 colonies per plate) and allowed to grow into colonies for 2 days. The colonies were then counted and the number of CFUs per plate calculated. Results were not statistically significant # p > 0.01.
